# A Socially Assistive Robot for Outpatient Eating Disorder Waiting‐List Support: A Mixed‐Methods Feasibility Randomised Controlled Trial

**DOI:** 10.1002/erv.70141

**Published:** 2026-06-26

**Authors:** Dimitri Chubinidze, Zhuo Li, Ayse Gundogan, Karina L. Allen, Petr Slovak, Kate Tchanturia

**Affiliations:** ^1^ Department of Psychological Medicine, King's College London Institute of Psychiatry, Psychology and Neuroscience (IoPPN) London UK; ^2^ South London and Maudsley NHS Foundation Trust National Eating Disorders Service London UK; ^3^ Department of Informatics King's College London London UK; ^4^ Department of Psychology Ilia State University Tbilisi Georgia; ^5^ Department of Culture and Communication, Faculty of Social Sciences and Humanities Aalborg University Aalborg Denmark

## Abstract

**Objective:**

This feasibility randomised controlled trial (RCT) evaluated Purrble, a socially assistive robot for emotion regulation, as a low‐intensity adjunct during waitlist periods in adult eating disorder services.

**Method:**

Adults waiting for outpatient eating disorder treatment were randomised to four weeks of Purrble use or waitlist‐as‐usual. The primary outcome was emotion regulation, assessed with the Difficulties in Emotion Regulation Scale; secondary outcomes included sensory sensitivity, anxiety, depression, and motivation for change. Analyses were conducted using paired *t*‐tests and linear mixed‐effects models. Acceptability was explored via a qualitative survey.

**Results:**

39 participants were enrolled in the trial and 37 participants were retained (19 Purrble, 18 control). A significant group × time interaction emerged for depression, with greater reductions in symptoms among Purrble participants. No significant interactions were found for emotion regulation, sensory sensitivity, motivation, or anxiety, though the Purrble group showed small‐to‐moderate improvements in emotion regulation, sensory sensitivity, and anxiety, and a significant within‐group increase in motivation. Qualitative findings identified four categories: situational use during distress, sensory comfort and emotional connection, design limitations/barriers, and continued use with integration into self‐care.

**Conclusions:**

Purrble proved acceptable and feasible as a waitlist adjunct in adult eating disorder outpatient services. A significant between‐group effect on depression and a within‐group improvement in motivation provide preliminary support for a fully powered trial.

## Highlights

- The socially assistive robot Purrble—a small, tactile companion device—could support patients on waiting lists for eating disorder treatment.
- In this feasibility trial, Purrble was positively evaluated by patients on a waiting list. Those using the device showed a reduction in depressive symptoms and a small increase in motivation.
- Purrble was used frequently. Participants described feelings of comfort, support with emotion regulation, and sensory soothing, and many continued using it after the study.

## 1 Introduction and Aims

Eating disorders (ED) are complex conditions associated with serious physical, psychological, and social burden (Treasure et al. 2020). Clinical services face significant pressure globally, with waiting lists remaining problematic where demand outstrips available resources (Wade et al. 2024). In the UK, mental healthcare is stratified by illness severity and individuals who do not require hospitalisation are referred to outpatient services (Garratt et al. 2024). Statutory access and waiting‐time standards apply to children and young people with ED, but the clinical reality in adult services is different (NHS England 2015; NHS Digital 2024; Beat 2019). While 79% of children and young people begin treatment within four weeks, adults often experience longer waits. An audit by Beat—the UK's largest eating disorders charity—reported a mean waiting time of 9 weeks, with only 26% of adults starting treatment within four weeks and some services reporting delays of up to 24 weeks or longer (Beat 2025). Furthermore, the duration of untreated ED is notably extended, averaging 2.5 years in anorexia nervosa (AN) and reaching 6 years in binge eating disorder (BED) (Austin et al. 2021).

These extended waiting times are clinically significant, as they are associated with increased illness severity, reduced motivation, and treatment disengagement (Carter et al. 2012; Solmi et al. 2024; Valentine et al. 2024). They also increase the risk of fragmented and less coordinated care, with some patients and families seeking support from informal or non‐specialist alternatives (Thomas et al. 2021). Given the high morbidity and mortality associated with ED, the lack of structured support during waiting periods is a pressing concern.

Individuals with ED often experience difficulties in emotion regulation and sensory processing, both of which contribute to symptom maintenance and poorer treatment outcomes (da Luz et al. 2023; Dingemans et al. 2017; Tchanturia 2022; Brockmeyer et al. 2014; Harrison et al. 2010; Wayda‐Zalewska et al. 2022). Heightened distress can precipitate disordered behaviours, while sensory sensitivities may exacerbate anxiety and overwhelm in everyday situations (da Luz et al. 2023; Kinnaird et al. 2020; Kinnaird and et al. 2021; Kinnaird et al. 2018; Merwin et al. 2013; Chubinidze, Zesch, et al. 2024; Leppanen et al. 2022). Motivation for change is another critical factor; it often fluctuates and may decrease during extended waits for treatment, but can also be enhanced through supportive interventions that foster readiness and self‐efficacy (Carter et al. 2012; Keegan et al. 2024). Therefore, emotion dysregulation, sensory sensitivity, and variable motivation represent important targets for support.

Interventions that provide immediate, in‐the‐moment strategies for self‐soothing and regulation may help stabilise these difficulties, sustain motivation, and enhance readiness to engage when specialist treatment becomes available. Recent randomised controlled trials and systematic reviews suggest that even minimal support, such as single‐session or brief psychoeducational interventions, may enhance nutritional health, strengthen self‐efficacy, and improve treatment retention (Wade et al. 2024; Keegan et al. 2024; Fursland et al. 2018). These studies demonstrate the feasibility and potential value of waitlist interventions that do not divert resources from frontline care. This has led to increasing interest in scalable digital and technology‐enhanced approaches, which can be deployed with minimal clinician input and are acceptable to patients (Rohrbach et al. 2023).

Within this landscape, digital health technologies (DHT)—technology‐enabled tools and platforms, including mobile applications, online programmes, wearable devices, and socially assistive robots, designed to support health assessment, monitoring, or intervention delivery—present promising mitigation strategy for improving access to clinical care. Previously, diverse DHT have shown favourable outcomes across different populations and clinical contexts. Fully automated online self‐help interventions for ED have produced significant reductions in eating disorder symptoms (Rohrbach et al. 2022; Sharp et al. 2025), and an automated conversational cognitive‐behavioural therapy (CBT) chatbot significantly reduced depressive symptoms compared to a control condition in young adults (Fitzpatrick et al. 2017). One domain of DHTs is socially assistive robots (SAR): physically embodied devices designed to provide social and emotional support through tactile interaction, distinguishing them from screen‐based applications and AI‐driven chatbots. SAR have produced favourable results in reducing distress and increasing positive affect in various populations, including improvements in loneliness and quality of life in older adults with dementia (Kabacińska et al. 2021; Petersen et al. 2016; Robinson et al. 2013) and significant reductions in anxiety and improvements in emotion regulation among highly anxious university students (Williams, Freed, et al. 2023). Unlike many online or AI‐based tools, which raise ongoing concerns around ethics and safeguarding (Chan et al. 2022), SAR are typically tangible, low‐intensity, and straightforward to implement in clinical settings. The device used in this study, Purrble, is a standalone interactive tactile companion designed to support emotion regulation (Slovák et al. 2018).

Purrble was originally developed by Slovák and colleagues at University College London and UC Santa Cruz as part of a research programme on socio‐technical interventions for families (Slovák et al. 2018) and has since been commercialised. It is a small, soft, furry creature‐like device with no screen, no artificial intelligence, and no internet connectivity. Drawing on Gross's extended process model of emotion regulation (Gross 2015), Purrble's design targets two stages of the regulation process: attentional deployment—redirecting attention away from the distressing situation towards interacting with the device—and response modulation—facilitating physiological down‐regulation through pleasant tactile interaction (Williams, Freed, et al. 2023; Williams et al. 2024). Purrble is framed as an anxious creature that requires soothing through gentle interaction. The device communicates its simulated ‘emotional state’ via an artificial heartbeat generated by internal vibrotactile electronics, producing vibrations that shift from rapid and agitated to steady and calm. The Purrble begins each interaction in an agitated state and calms progressively as the user engages through gentle physical contact such as stroking or holding. The underlying logic model operates at three levels: (1) providing in‐the‐moment soothing by shifting attention and modulating arousal; (2) facilitating sustained engagement through the relational framing of caring for the device; and (3) over time, promoting a shift in emotion regulation practices and implicit beliefs about the controllability of one's emotions (Williams et al. 2024). A detailed description of Purrble's logic model is provided in Williams et al. (2024, Supplementary Material 1). Initially developed for children, Purrble has demonstrated positive results in different adult populations, including highly anxious university students and ethnic minority youth at risk of self‐harm (Williams, Freed, et al. 2023; Slovák et al. 2018; Theofanopoulou et al. 2019; Williams, Townsend, et al. 2023).

The pilot work in ED services has demonstrated Purrble's acceptability and engagement, with patients reporting its integration into self‐soothing and sensory regulation routines (Chubinidze, Li, et al. 2024). A qualitative pilot study with ED and PTSD comorbidity highlighted its perceived value for calming nightmares, reducing overstimulation, and providing emotional comfort (Chubinidze et al. 2025). Outside ED services, an open trial with highly anxious community sample reported significant within‐group reductions in anxiety and improvements in emotion regulation over a 2‐week period (Williams, Freed, et al. 2023), and ongoing RCTs in youth at risk of self‐harm have shown sustained engagement (Williams et al. 2024).

Building on this groundwork, the present study reports a mixed‐methods feasibility RCT of Purrble, a socially assistive self‐soothing device, as a low‐intensity adjunct during the waitlist phase in an adult specialist outpatient ED service. The primary aim was to evaluate feasibility and acceptability, with exploratory analyses of emotion regulation, sensory sensitivity, mood, and motivation to inform the design of a future fully powered trial.

## 2 Method

### 2.1 Study Design

This study was a single‐site, two‐arm, parallel‐group feasibility randomised controlled trial (RCT). Participants were allocated to either the intervention group (receiving Purrble) or the control group (waitlist‐as‐usual only). As an ethical provision, control participants received a Purrble device after the 4‐week intervention period ended, so that all participants could benefit from the intervention; no crossover was implemented during the trial itself. The intervention period lasted four weeks, with self‐report outcomes collected weekly during the intervention and followed up 8 weeks post‐intervention via an online feedback form. The study was intended to assess feasibility and acceptability, and to generate preliminary outcome data to inform future research. This is consistent with guidance on feasibility trial methodology, which suggests that samples of approximately 24–50 participants are appropriate for estimating recruitment, retention, and preliminary effect sizes to inform a fully powered trial (Eldridge et al. 2016).

Of note, both groups remained on the ED service waitlist and could access waitlist support as provided in routine care. This could include written psychoeducational materials on ED symptoms and change strategies, monthly online psychoeducational workshops on rotating topics, and signposting to external support resources such as the Beat eating disorder charity (beateatingdisorders.org.uk), the FREED early intervention programme (firststepsed.co.uk), the PEACE pathway for ED and autism (peacepathway.org), and other online self‐help resources. Access to these supports varied between participants and were not standardised within the trial. Because support use was recorded as part of routine clinical care rather than collected systematically for research purposes, it was not possible to reliably quantify or control for these factors in the present study.

### 2.2 Participants and Recruitment

Participants were recruited from the adult specialist ED outpatient service of the South London and Maudsley NHS Foundation Trust. Patients who were assessed and given an ED diagnosis, placed on the outpatient waitlist for treatment, and who had provided Consent for Contact (C4C) for research studies were approached via email. The email provided a flyer with a brief study description and invited patients to register their interest in participating. Screening was conducted against predefined inclusion and exclusion criteria (Table 1), using an online screening form with verification with clinical records. Written informed consent was obtained electronically before enrolment.

**TABLE 1:** Summary of inclusion and exclusion criteria.

Inclusion criteria	Exclusion criteria
Currently on the SLaM ED outpatient service waiting list	Current substance use problems
Confirmed DSM‐5 eating disorder diagnosis	Active psychosis
Aged 18 years or older	Current high suicidality or high risk of self‐harm
Willing to commit to the study and provide informed consent	Current engagement in another ED treatment
	Previous exposure to the Purrble intervention

Abbreviations: DSM‐5, Diagnostic and Statistical Manual of Mental Disorders, Fifth Edition; ED, eating disorder; SLaM, South London and Maudsley NHS Foundation Trust.

### 2.3 Randomisation and Allocation

Following consent, participants were anonymised and allocated to trial arms using simple randomisation in a 1:1 ratio. The randomisation sequence was generated with a computer‐based random number algorithm in Microsoft Excel. The researcher who created the randomisation sequence (JL) was blinded to allocation. Given the nature of the intervention, participants and outcome assessors were not blinded.

### 2.4 Intervention

Participants allocated to the intervention group received a Purrble device in addition to waitlist‐as‐usual support. Devices were posted to participants' home addresses. Each device was accompanied by a user manual. Participants were encouraged to engage with the device freely, particularly during periods of heightened emotional distress or sensory overload. No minimum or maximum frequency of use was prescribed. After the intervention period, all participants were allowed to keep the device.

### 2.5 Data Collection and Outcomes

Baseline data were collected during the second week following randomisation (the pre‐deployment phase), using an online Microsoft Forms questionnaire, which was also used for all subsequent assessments. Week 1 was used for consent confirmation, randomisation, and postal dispatch of the Purrble device; baseline assessments were therefore scheduled in Week 2 to ensure completion before device receipt. Participants were asked to complete weekly assessments during the 4‐week intervention period and at post‐intervention follow‐up. An overview of all outcome measures and their corresponding assessment time points is provided in Table 2.

**TABLE 2:** Outcome measures and timing of data collection.

Outcome measure	Group	*T*0 (Pre‐deployment, week 2)	*T*1 (week 3)	*T*2 (week 4)	*T*3 (week 5)	*T*4 (week 6)	Follow‐up (week 14)
Difficulties in emotion regulation Scale‐8 (DERS‐8)	Purrble	✓	✓	✓	✓	✓	—
	Control	✓	✓	✓	✓	✓	—
Glasgow sensory questionnaire short version (GSQ‐14)	Purrble	✓	✓	✓	✓	✓	—
	Control	✓	✓	✓	✓	✓	—
Hospital anxiety and Depression scale (HADS)	Purrble	✓	✓	✓	✓	✓	—
	Control	✓	✓	✓	✓	✓	—
Motivational Ruler	Purrble	✓	✓	✓	✓	✓	—
	Control	✓	✓	✓	✓	✓	—
Autism spectrum quotient Short version (AQ‐10)	Purrble	✓	—	—	—	✓	—
	Control	✓	—	—	—	✓	—
Twente Engagement With eHealth Technologies Scale (TWEETS)	Purrble	✓	✓	✓	✓	✓	—
	Control	—	—	—	—	—	—
Purrble engagement questions: 1. How many times have you used the Purrble this week? 2. In what situations did you tend to use it?	Purrble	✓	✓	✓	✓	✓	—
	Control	—	—	—	—	—	—
Feedback form	Purrble	—	—	—	—	—	✓
	Control	—	—	—	—	—	✓

*Note: T*0 = Week 2 (pre‐deployment baseline); *T*1 = Week 3; *T*2 = Week 4; *T*3 = Week 5; *T*4 = Week 6 (end of intervention).

The primary outcome was emotion regulation, assessed using the Difficulties in Emotion Regulation Scale–Short Form (DERS‐8). This measure provides a unidimensional assessment of emotion regulation difficulties, with total scores ranging from 8 to 40 (Penner et al. 2023). The short form was selected to minimise participant burden while retaining robust psychometric properties The DERS‐8 showed good internal consistency in this sample (Cronbach's *α* = 0.90).

Secondary outcomes included:

Sensory sensitivity, assessed with the Glasgow Sensory Questionnaire—Short (GSQ‐14), which measures both hypo‐ and hyper‐reactivity across seven sensory modalities (score range: 0–56) (Millington and Simmons 2023).

Anxiety and depression, assessed with the Hospital Anxiety and Depression Scale (HADS), a widely used clinical measure with anxiety and depression subscales (score range per subscale: 0–21; clinical cut‐offs: 0–7 normal, 8–10 mild, 11–14 moderate, 15–21 severe; Cronbach's *α*: anxiety = 0.77, depression = 0.76) (Zigmond and Snaith 1983).

Motivation and self‐efficacy, assessed with the Motivational Ruler (Miller and Rollnick 2012), a brief tool developed within motivational interviewing to measure readiness and confidence for change (score range: 0–20).

Exploratory outcomes were included to examine feasibility in subgroups and to assess engagement with the intervention. The Autism Spectrum Quotient‐10 (AQ‐10) (Allison et al. 2012) was used to screen for autistic traits (with ≥ 6 as the threshold). Engagement with and acceptability of the device were assessed using an adapted version of the Twente Engagement with eHealth Technologies Scale (TWEETS) (Kelders et al. 2020), a 9‐item self‐report measure on a 0‐4 scale with three subscales (behaviour, cognition, affect) (Cronbach's *α* = 0.84). Of note, Purrble does not have built‐in usage logging or data export capability; self‐reported weekly engagement questions were therefore used as a proxy for device use frequency.

Qualitative feedback was obtained from all participants through a short follow‐up questionnaire containing six open‐ended questions on use contexts, perceived value, reflections, improvement suggestions, and future intentions (see Table S1 for full questions).

### 2.6 Data Analysis

Quantitative data were analysed using Stata Statistical Software (Release 18) (StataCorp 2023) and IBM SPSS Statistics (Version 31) (IBM Corp 2025). Given the feasibility design and limited sample size, analyses were exploratory, prioritising clarity and interpretability over hypothesis testing. Within‐group changes were examined using paired‐sample *t*‐tests, and between‐group differences at baseline were assessed using two‐sample *t*‐tests. Linear mixed‐effects models were employed to explore changes over time and group × time interactions, adjusting for baseline BMI, illness duration, and AQ‐10. Effect sizes (Cohen's d) were also reported. No corrections were applied for multiple comparisons, consistent with recommendations for exploratory feasibility analysis (Eldridge et al. 2016).

Qualitative data were analysed using an inductive content analysis approach (Graneheim and Lundman 2004; Elo and Kyngäs 2008). Responses were coded manually, and recurrent patterns were collated into themes reflecting participants' experiences of the intervention. Coding was conducted by the first author (DC), with cross‐checking and discussion within the research team to enhance rigour.

Quantitative and qualitative findings were subsequently triangulated. Convergence and divergence across data strands were examined, with qualitative themes used to provide explanatory context for quantitative signals.

### 2.7 Trial Registration and Ethical Approval

The trial was registered with clinicaltrials.gov (Identifier: NCT07007481). Ethical approval was granted by the London–Surrey Borders Research Ethics Committee (REC reference: 24/LO/0100).

## 3 Results

### 3.1 Participant Flow and Baseline Characteristics

The participant enrolment and allocation process is summarised in the CONSORT flow diagram (Figure 1). A total of 39 participants were enrolled from the outpatient ED waiting list and randomised to either the Purrble group (*n* = 20) or waitlist‐as‐usual (*n* = 19). Two participants withdrew for reasons unrelated to the intervention—one due to referral to a different ED service and one due to family‐related travel—(one from each arm), leaving 19 in the Purrble group and 18 in the control group. All remaining participants completed outcome measures at each time point. All remaining participants completed outcome measures at each time point.

**FIGURE 1:**
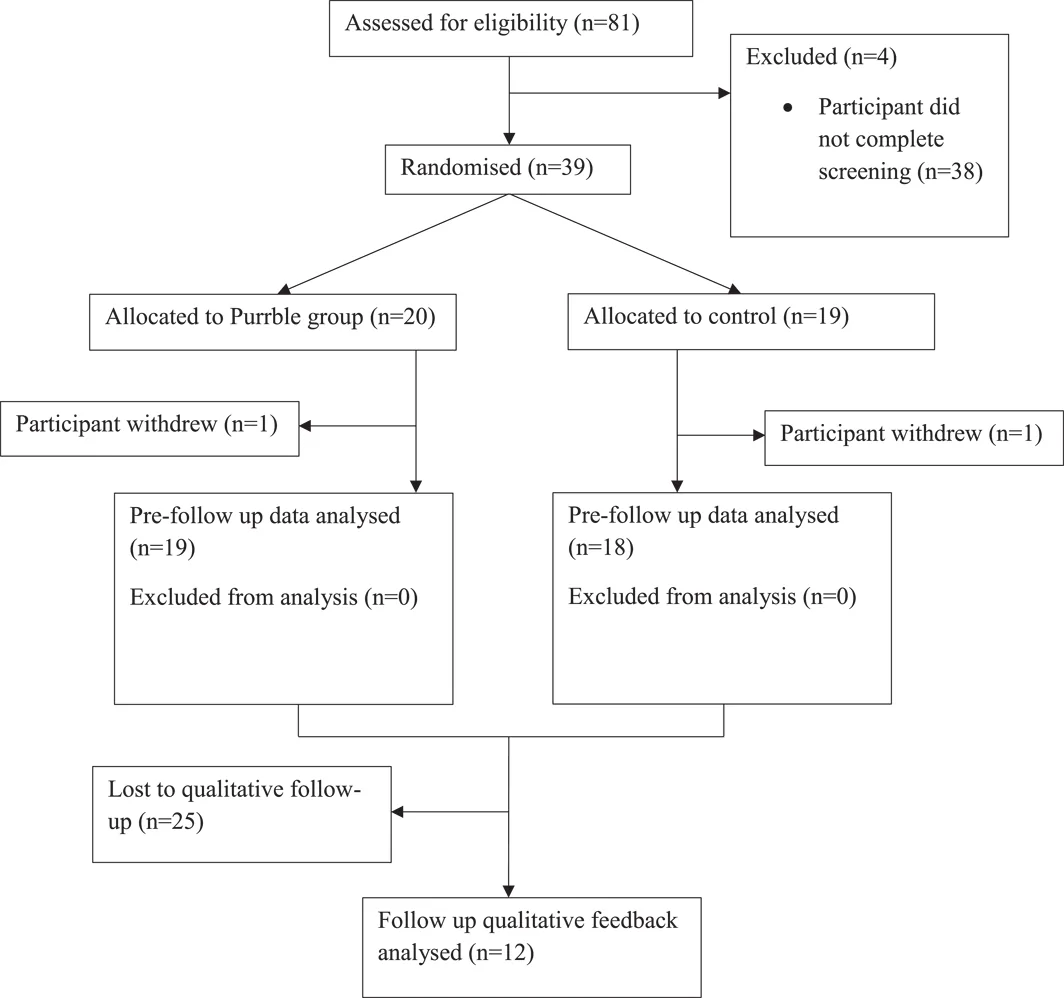
CONSORT flow diagram for participant recruitment, allocation, and follow‐up.

After completion of the intervention period, all participants (from both arms) were invited to complete an optional qualitative follow‐up survey distributed 2 months post‐intervention. As described in the Study Design, control participants received a Purrble device after the intervention period concluded; the qualitative survey was therefore distributed after both groups had used the device. Twelve participants (32%) returned the survey. Because all participants had used Purrble by this point, qualitative data were analysed across the combined sample rather than by randomisation arm.

Participants' baseline demographics are summarised in Table 3. Seventeen participants (45.9%) scored above the AQ‐10 threshold for high autistic traits. There were no significant differences between the Purrble and control groups for any of the characteristics measured (all *p* > 0.05) based on *t*‐tests and Fisher exact tests.

**TABLE 3:** Baseline demographics.

Demographics	Intervention group (*n* = 19)	Control group (*n* = 18)
Age (years), mean (SD)	32.0 (10.8)	37.1 (12.1)
Female, *n* (%)	16 (84.2%)	17 (94.4%)
Illness duration (years), mean (SD)	15.8 (9.6)	20.7 (13.1)
Psychotropic medication, *n* (%)	5 (26.3%)	6 (33.3%)
BMI, mean (SD)	23.28 (10.31)	20.97 (5.07)
Ethnicity, *n* (%)		
White British	14 (73.7%)	13 (72.2%)
White Other	3 (15.8%)	1 (5.6%)
Black British	1 (5.3%)	2 (11.1%)
Asian	1 (5.3%)	1 (5.6%)
Mixed	0	1 (5.6%)

Abbreviation: SD, standard deviation.

### 3.2 Primary and Secondary Outcomes

Primary and secondary outcomes for the Purrble (*n* = 19) and control (*n* = 18) groups across all timepoints are outlined in Table 4. There were no significant between‐group differences at baseline for any outcomes (all *p* > 0.05).

**TABLE 4:** Primary and secondary outcomes for the Purrble and control groups over all timepoints.

Outcome measures	*T*0, mean (SD)	*T*1, mean (SD)	*T*2, mean (SD)	*T*3, mean (SD)	*T*4, mean (SD)
DERS‐8^a^					
Purrble	30.6 (6.7)	28.0 (5.0)	28.3 (4.5)	29.1 (5.5)	28.6 (6.3)
Control	31.5 (7.0)	31.9 (6.8)	31.5 (6.7)	32.8 (7.1)	31.4 (8.6)
GSQ‐14^b^					
Purrble	19.2 (10.1)	18.9 (9.4)	17.3 (10.4)	15.9 (9.0)	16.5 (10.1)
Control	21.9 (10.5)	23.4 (12.1)	21.6 (13.1)	22.6 (13.4)	22.8 (13.5)
Motivation					
Purrble	12.1 (1.5)	12.8 (2.8)	12.9 (3.0)	13.0 (2.9)	13.6 (3.0)
Control	12.9 (2.4)	12.6 (2.7)	12.4 (2.0)	13.2 (2.3)	12.8 (2.7)
HADS^c^ anxiety					
Purrble	15.5 (3.7)	15.1 (2.9)	14.6 (3.0)	14.6 (3.7)	14.1 (3.9)
Control	16.1 (3.9)	16.1 (3.8)	15.8 (4.0)	15.6 (4.2)	15.3 (5.0)
HADS Depression					
Purrble	9.9 (4.0)	9.4 (4.5)	9.2 (4.3)	9.3 (4.2)	9.1 (4.7)
Control	11.1 (4.5)	11.9 (5.1)	12.6 (4.7)	12.2 (4.9)	11.9 (5.0)

*Note: T*, time point. *T*0 = Week 2 (pre‐deployment baseline); *T*1 = Week 3; *T*2 = Week 4; *T*3 = Week 5; *T*4 = Week 6 (end of intervention). HADS clinical cut‐offs per subscale: 0–7, normal; 8–10, mild; 11–14, moderate; 15–21, severe.

Abbreviations: DERS, Difficulties in Emotion Regulation Scale; GSQ, Glasgow Sensory Questionnaire; HADS, Hospital Anxiety and Depression Scale.

^a^DERS‐8 = 8‐item Difficulties in Emotion Regulation Scale.

^b^ GSQ‐14 = 14‐item Glasgow Sensory Questionnaire.

^c^ HADS anxiety = Hospital Anxiety Scale anxiety subscale.

### 3.3 Emotion Regulation

The linear mixed‐effects model revealed no significant group × time interaction (*b* = −0.36, *p* = 0.422), indicating that changes in emotion regulation over time did not differ significantly between the Purrble and control groups. Within‐group analyses showed a small, non‐significant improvement in the Purrble group from baseline to *T*4 (*p* = 0.246, *d* = 0.28), with a negligible change in the control group (*p* = 0.966, *d* = 0.01).

### 3.4 Sensory Sensitivity

The model examining sensory sensitivity showed no significant overall group × time interaction (*b* = −0.94, *p* = 0.070), though the trend suggested a greater reduction in sensory sensitivity over time for the Purrble group compared to controls. Within‐group analyses indicated that the change in GSQ from baseline to *T*4 was not significant for either group; however, the Purrble group showed a small‐to‐moderate effect size (*p* = 0.134, *d* = 0.36) compared to a negligible effect in the control group (*p* = 0.541, *d* = 0.15).

### 3.5 Motivation

The group × time interaction was not significant (*b* = 0.31, *p* = 0.155), suggesting that changes in motivation over time did not differ significantly between the Purrble and control groups. Within‐group analyses revealed a significant improvement in motivation for the Purrble group from baseline to *T*4 (*p* = 0.039, *d* = 0.51), with a medium effect size. In contrast, the control group showed no significant change (*p* = 0.801, *d* = 0.06).

### 3.6 Anxiety

The linear mixed‐effects model revealed no significant group × time interaction (*b* = −0.13, *p* = 0.544), indicating that changes in anxiety over time did not differ significantly between the Purrble and control groups. Within‐group analyses showed that anxiety scores decreased slightly but not significantly for the Purrble group (*p* = 0.091, *d* = 0.41), with a moderate effect size. Similarly, the control group also showed a non‐significant reduction in anxiety (*p* = 0.159, *d* = 0.35).

### 3.7 Depression

The model showed a significant group × time interaction (*b* = −0.38, *p* = 0.043), indicating that the trajectory of depression scores over time differed between the Purrble and control groups, with a greater reduction observed in the Purrble group. Within‐group analyses indicated modest, non‐significant decreases in depression scores for both the Purrble group (*p* = 0.145, *d* = 0.35) and control group (*p* = 0.195, *d* = 0.32).

### 3.8 Engagement

Among Purrble arm participants who completed the Twente Engagement with eHealth Technologies Scale (*n* = 19), the mean total score was 22.4 out of 36, indicating a moderate level of engagement with Purrble. Subscale scores were similar for behavioural (7.4), cognitive (7.2), and affective (7.8) engagement, with the highest mean observed for affective engagement, suggesting positive emotional responses towards using Purrble.

### 3.9 Qualitative Feedback

Twelve participants completed the follow‐up survey (Table S1). Responses were analysed by DC using inductive qualitative content analysis. An initial set of 18 codes was generated through line‐by‐line coding, which were subsequently grouped into four categories through iterative discussion within the research team. Illustrative quotes for each category are presented in Table 5.

**TABLE 5:** Qualitative categories and illustrative participant quotes on experiences with the Purrble device.

Categories	Description	Illustrative quote
Situational use during emotional distress	Purrble was used in moments of anxiety, stress, or dysregulation	‘When feeling very overwhelmed and anxious’. (P9)
		‘When anxious and usually when alone’. (P5)
		‘When I felt sad/low/alone’ (P10)
Sensory comfort and emotional connection	Participants valued tactile features for calming and emotional support	‘The softness and vibrations’. (P1)
		‘The soothing feel of the heartbeat’. (P12)
		‘The calming influence the sensation of stroking its fur and how it changed the pace of its breath/made it purr’. (P9)
Design limitations and barriers to use	Suggestions included making Purrble less childlike, softer, or more adjustable	‘The on/off switch is a bit awkward to access’. (P2)
		‘It feels a little bit juvenile’. (P8)
		‘I think if it were a bit bigger it would have a broader impact. But the size it currently is makes it great to travel with. Perhaps creating a larger one to complement the smaller one would be good. That would enable it to be a bit softer and squishier too. More of a sense of being hugged and soothed’. (P5)
Continued use and self‐care integration	Several planned to keep using Purrble as part of self‐care routines	‘I have been using it since the trial ended. It's a remarkable innovation’. (P5)
		‘Yes, in situations of anxiety, stress, sadness or overwhelm’. (P12)

#### 3.9.1 Situational Use During Emotional Distress

All 12 participants described using Purrble during periods of heightened emotional arousal—most commonly anxiety, overwhelm, and stress. Most reported reactive use, reaching for the device when distress was rising, with several describing use at bedtime or during evening hours: ‘Whenever I could feel my anxiety start to increase’ (P11); ‘When anxious and usually when alone’ (P5). One participant described using Purrble when feeling ‘sad/low/alone’ (P10), suggesting it served a function across both anxious and low‐mood states. The consistency of these accounts indicates that Purrble was readily adopted as an in‐the‐moment tool for managing emotional difficulty.

#### 3.9.2 Sensory Comfort and Emotional Connection

Participants identified Purrble's tactile and sensory qualities—its soft texture, vibrations, and simulated heartbeat—as its most valued features. These were described as physically calming and as fostering a sense of emotional connection: ‘Soft, calm and friend’ (P11); ‘The soothing feel of the heartbeat’ (P12). Several participants highlighted the contingent feedback between their touch and Purrble's response: ‘The calming influence the sensation of stroking its fur and how it changed the pace of its breath/made it purr’ (P9). One participant described Purrble as functioning as both a distraction and a regulatory aid: ‘How it responds to your interaction distracting you from what you're feeling, in turn helping you calm down’ (P3). Another reflected that using the device retrospectively revealed the extent of their distress: ‘Sometimes I couldn't identify how anxious I had been feeling until after I'd used it’ (P5).

#### 3.9.3 Design Limitations and Barriers to Use

While feedback was predominantly positive, several participants identified design‐related barriers. Concerns centred on Purrble's appearance being perceived as ‘juvenile’ (P8) or ‘childish’ (P10), with suggestions for a more discreet design (P4) or additional design variants to broaden accessibility (P8). Physical modifications were also proposed, including increased softness (P9, P12), a larger version for home use alongside the current portable size (P5), and stronger purring vibrations (P11). One participant with a more complex mental health presentation felt the device was insufficient for their needs: ‘My needs are beyond a Purrble’ (P10). This was the only participant to express a wholly negative view, and their feedback highlights that while the intervention may be broadly acceptable, it may not meet the needs of individuals with more complex presentations.

#### 3.9.4 Continued Use and Self‐Care Integration

Ten of the 12 respondents expressed an intention to continue using Purrble beyond the trial, with several describing the device as already embedded in their self‐care routines. One participant, who had named the device, said: ‘I have never stopped using Zazu’ (P11), suggesting a degree of relational attachment. Another described integrating Purrble into their recovery: ‘I have been using it since the trial ended. It's a remarkable innovation. I would like to better incorporate it into my self‐care routine. I think as I go on my weight gain journey, I'll need to have those kinds of measures in place’ (P5). One participant planned to extend use beyond emotion regulation to managing disordered eating urges: ‘I plan to try to make more use of it to help calm my mind before going to sleep and also try using it as a distraction tool when disordered urges arise’ (P9). Two participants did not intend to continue: one citing uncertainty due to the device feeling juvenile (P8), and one expressing that it did not meet their needs (P10).

When asked to describe their experience with Purrble in three words, 10 of the 12 participants (83%) used positive emotional descriptors, with terms such as ‘calming,’ ‘soothing,’ ‘comforting,’ and ‘relaxing’ appearing most frequently. Examples included ‘soft, calm and friend’ (P11) and ‘calming, grounding, content’ (P12). One participant offered an exclusively negative response: ‘Disappointing. Childish. No use’ (P10). Illustrative quotes for each category are presented in Table 5.

## 4 Discussion

This feasibility study evaluated Purrble—a socially assistive robot designed to support emotion regulation—as a low‐intensity adjunct for adults on the waiting list of a specialist outpatient ED service. The trial was designed to assess feasibility and acceptability and to generate preliminary signals of device utility for emotion regulation, sensory sensitivity, mood, and motivation in this population. Effect sizes and within‐group changes are therefore reported as preliminary estimates to inform a future definitive trial. Given reported delays in access to ED treatment in the UK and internationally (Wade et al. 2024; Beat 2025; Austin et al. 2021), these findings contribute to a growing body of literature on the need for scalable, low‐intensity interventions to bridge gaps in care.

Recruitment, retention, and outcome completion rates support the feasibility of delivering Purrble in this setting. Of 39 participants randomised, 37 were retained (95%), and outcome measure completion was high across all assessment time points. Engagement scores indicated moderate uptake of the device, with the highest mean on the affective subscale, and qualitative accounts described situational use during distress, sensory comfort, and integration into self‐care routines. Together, these findings indicate that Purrble can be delivered to adults waiting for ED treatment. Across primary and secondary outcomes, no significant group × time interactions emerged for emotion regulation, sensory sensitivity, motivation, or anxiety, although the Purrble group showed small‐to‐moderate within‐group improvements across these domains. A significant group × time interaction was observed for depression, indicating a greater reduction in depressive symptoms among Purrble participants relative to controls, alongside a significant within‐group increase in motivation in the Purrble group.

Within the intervention group, trends towards reduced psychological distress and improved motivation are consistent with the broader emerging literature on waitlist interventions in ED care. Even minimal structured support during waiting periods has been shown to influence treatment readiness and agency in help‐seeking (Wade et al. 2024; Keegan et al. 2024). A recent RCT of a brief waitlist intervention reported improved treatment retention, suggesting that low‐intensity support during the pre‐treatment period can have downstream clinical value (Keegan et al. 2024). A single‐session assessment and psychoeducational intervention for ED also demonstrated positive effects on waitlist outcomes (Fursland et al. 2018), and a chatbot‐based single‐session intervention showed promise in reducing ED symptoms among waitlisted individuals (Sharp et al. 2025). These findings sit within a broader shift towards developing structured waitlist solutions in ED care, as outlined by Wade et al. 2024, who argue that even brief, scalable interventions can meaningfully address the clinical risks associated with prolonged treatment delays. The within‐group motivation improvement observed here (*d* = 0.51) is consistent with this evidence, although the absence of a significant between‐group interaction means it cannot be attributed specifically to the device.

The significant group × time interaction for depression indicates that depression trajectories differed between groups across the intervention period, with greater reductions among Purrble participants. Data presented in the Table 4 suggests this was driven by a steady decrease in HADS‐Depression scores in the Purrble group alongside an initial increase and partial return to baseline in the control group, rather than by a large baseline‐to‐*T*4 difference in either arm. While this pattern should be interpreted with caution given the feasibility design, it is consistent with Purrble's proposed mechanisms of attentional deployment, response modulation, and sustained affiliative engagement (Gross 2015; Williams et al. 2024). Comparable directional findings have been reported in related literature: Robinson et al. 2013 observed improvements in loneliness—closely associated with depressive symptoms—in a companion robot trial with older adults, and Williams, Freed, et al. (2023) reported within‐group reductions in anxiety using Purrble with highly anxious university students. The convergence of direction across these populations and our study suggests a plausible shared mechanism, which warrants further investigation in adequately powered trials with active control conditions.

The pattern of effects across anxiety and depression also warrants consideration. The within‐group effect size for anxiety reduction in the Purrble group (*d* = 0.41) was somewhat larger than for depression (*d* = 0.35), yet the significant group × time interaction emerged for depression rather than anxiety. One possible explanation is that Purrble's response modulation function—providing immediate sensory soothing through tactile engagement—may target anxious arousal directly, producing within‐group change in both arms (the control group also showed a non‐significant anxiety reduction, *d* = 0.35). Depressive symptoms, by contrast, may respond more gradually through sustained affiliative engagement, potentially differentiating Purrble users from controls over time. The waitlist period itself is also likely to generate anticipatory anxiety in both groups, which may have attenuated between‐group differences for anxiety specifically. Disentangling device‐specific from context‐related effects will require an active control condition and longer follow‐up in a future study.

Several alternative explanations can be considered. It remains unclear whether the observed changes reflect specific effects of Purrble or more general study‐related factors, such as increased engagement, perceived support, and regular contact with researchers. The within‐group motivation finding warrants particular caution: while motivation predicts treatment outcomes in EDs, its predictive value varies across symptom domains (Vall and Wade 2015), and in the absence of a significant between‐group interaction, the improvement may reflect non‐specific effects of trial participation. The high proportion of participants scoring above the AQ‐10 threshold for autistic traits (46%) is also relevant: emotion regulation and sensory profiles differ across neurotypes (Tchanturia 2022; Kinnaird et al. 2020; Kinnaird and et al. 2021), and it is possible that Purrble's tactile, low‐demand mode of engagement is particularly well‐suited to neurodivergent participants—a hypothesis that formal testing. Qualitative findings complemented the quantitative trends, with participants describing situational use during distress, calming sensory qualities, and integration into self‐care strategies. This is consistent with meta‐analytic evidence that emotion regulation plays a central but heterogeneous role across ED presentations (Leppanen et al. 2022), suggesting that interventions targeting regulation—including sensory‐based approaches—warrant further investigation across diagnostic and neurodiverse subgroups.

### 4.1 Strengths and Limitations

The study has several strengths. It was embedded in a specialist clinical outpatient service where patients need to wait for treatment, increasing the ecological validity of the findings. The mixed‐methods design allowed quantitative patterns to be interpreted in light of participants' experiences, and brief, validated measures balanced methodological rigour with the practical demands of overstretched clinical services. Retention was high (95%), and outcome measure completion was acceptable across all time points.

Several limitations should be acknowledged. The small sample size limits statistical power, and the demographic profile—predominantly White British and female—restricts generalisability. Detailed diagnostic subtype and illness severity data were not consistently available, particularly for participants still undergoing service triage while on the waiting list, which limited subgroup analyses (Wayda‐Zalewska et al. 2022). Although workshop attendance was recorded by the service, individual‐level data were not incorporated, meaning that differential uptake of other interventions could not be controlled for. Treatment engagement and retention after the waiting period were not tracked, leaving open the question of whether early motivational or emotional gains translate into improved treatment uptake. Finally, the intervention was deliberately positioned as a low‐intensity adjunct targeting emotion regulation and self‐soothing and not ED psychopathology directly, which limits conclusions about disorder‐specific outcomes.

### 4.2 Implications and Future Directions

Preliminary signals of improved motivation and reduced distress suggest that low‐intensity, sensory‐attuned tools such as Purrble may help sustain engagement and emotional readiness during long waits for treatment. In health systems facing systemic backlogs and resource constraints, even modest gains in engagement, readiness, or emotional support could contribute to reducing the risk of disengagement.

Future trials should employ larger, actively controlled designs with longer‐term follow‐up to disentangle device‐specific from general participation effects, and to examine whether short‐term motivational or emotional gains influence subsequent treatment uptake and outcomes. Recruitment should explicitly target ethnically diverse samples to explore the effectiveness of SAR across cultural contexts. Stratifying by ED subtype and comorbidity—particularly neurodivergent groups (e.g., autism, ADHD)—will be important given the heterogeneity of emotion regulation and sensory profiles in ED populations; the high rate of autistic traits observed in the present sample further supports this priority. Such work would provide stronger evidence for the integration of socially assistive tools into stepped‐care models and waitlist support strategies for adult ED services.

## 5 Conclusion

This mixed‐methods feasibility RCT supports the acceptability and feasibility of Purrble as a waitlist adjunct in adult outpatient ED services. Preliminary signals—a between‐group effect on depression, a within‐group improvement in motivation, and qualitative accounts of sensory comfort and integration into self‐care—provide an early rationale for a fully powered trial of scalable, non‐invasive supports to address the care gap created by prolonged treatment delays.

## Funding

This study was funded by the Engineering and Physical Sciences Research Council (EPSRC) Impact Acceleration Account (IAA) EP/X525571/1; NIHR BRC for Mental Health, SLaM, and the Institute of Psychiatry, Psychology and Neuroscience, KCL; Medical Research Foundation (Changing Policy and Practice Award; Ref: MRF‐CPP‐R1‐25‐103); and the One King's Impact Fund and aligns with King's Impact Priorities: Whole‐life health for mind and body & Technology for good. KT is supported by UK Research and Innovation (MRC, ESRC, AHRC), the National Institute for 516 Health and Care Research and the Medical Research Foundation as part of the EDAC network (Grant 517 number: MR/X03058X/1). The views expressed are those of the authors and not necessarily those of the NIHR or the Department of Health and Social Care.

## Conflicts of Interest

The authors declare no conflicts of interest.

## Supporting information


**Table S1:** Open‐ended follow‐up survey questions.

## Data Availability

The data that support the findings of this study are available on request from the corresponding author. The data are not publicly available due to privacy or ethical restrictions.
