# Supplementary material for: A Socially Assistive Robot for Outpatient Eating Disorder Waiting‐List Support: A Mixed‐Methods Feasibility Randomised Controlled Trial
Source: Eur Eat Disord Rev. 2026 Jun 26;34(6):1562–73. doi: 10.1002/erv.70141 (PMC13656759; doi:10.1002/erv.70141)
Supplement: Supplementary file 1 [file ERV-34-1562-s001.docx]

**Supplementary Table S1.** Open-ended follow-up survey questions

| **Question** | **Question text** |
| --- | --- |
| 1 | In what situations did you tend to use Purrble? |
| 2 | What did you like the most about Purrble? |
| 3 | If you were to describe your experience with Purrble in three words, which words would you choose? (You may write single words or short phrases) |
| 4 | How could Purrble be improved to better meet your needs? |
| 5 | Do you see yourself using Purrble in the future? If so, how do you plan to use it? |
| 6 | Any other comments? |
